# “We Were Among the First Non-traditional Families”: Thematic Perceptions of Lesbian Parenting After 25 Years

**DOI:** 10.3389/fpsyg.2019.02414

**Published:** 2019-10-25

**Authors:** Nanette Gartrell, Esther D. Rothblum, Audrey S. Koh, Gabriël van Beusekom, Henny Bos

**Affiliations:** ^1^Williams Institute, UCLA School of Law, Los Angeles, CA, United States; ^2^Research Institute of Child Development and Education, University of Amsterdam, Amsterdam, Netherlands; ^3^Department of Women’s Studies, San Diego State University, San Diego, CA, United States; ^4^School of Medicine, University of California, San Francisco, San Francisco, CA, United States; ^5^Netherlands Institute for Social Research, The Hague, Netherlands

**Keywords:** lesbian parenting, sexual minority parent families, parent perspectives, thematic perceptions, same-sex parenting, emerging adults, benefits of same-sex parenting, challenges of same-sex parenting

## Abstract

In the sixth wave of the U.S. National Longitudinal Lesbian Family Study (NLLFS), when their offspring were 25 years old, the parents were asked to reflect on their most challenging and best experiences raising children in non-traditional families. The responses of 131 parents were interpreted through thematic analysis. The most challenging parenting experiences fell into five major categories: (1) distress about their children’s experiences of exclusion, heterosexism, or homophobic stigmatization; (2) family of origin non-acceptance of their lesbian-parent family; (3) the never-ending process of “educating the world about queer parents”; (4) homophobia or hostility toward their non-traditional family; and (5) lack of legal protections for sexual minority parent (SMP) families. Their best parenting experiences included: (1) being role models, leading to a greater acceptance of LGBTQ people; (2) treasuring the LGBTQ parent and family community; (3) teaching their children to appreciate diversity of all types; and (4) witnessing their child’s pride in their non-traditional family. Some of these challenges were anticipated by the parents more than a quarter century ago at the time that they were inseminating or pregnant with the index offspring.

## Introduction

“In third grade, I overheard <my child> telling his friends, ‘there are three unusual things about me: I don’t like chocolate, I don’t eat meat, and I have two moms.’ His friends, horrified, said, ‘YOU DON’T LIKE CHOCOLATE?!”’ recalled a parent in describing her best experiences.

Research on sexual minority parents (SMPs) has examined many aspects of parenthood, from the decisions they make in planning their families (Gartrell et al., 1996; Goldberg and Sayer, 2006; Goldberg, 2010), to their negotiations concerning the division of labor and parenting roles (Gartrell et al., 1999; Goldberg and Perry-Jenkins, 2007). Studies have reported on their sources of social support, their concerns about homophobic stigmatization, and their feelings about raising children without fathers or without mothers (Gartrell et al., 1996, 1999, 2000, 2006; Goldberg, 2010). There are data on single parents, couple dynamics, and parental relationship dissolution among those who identify as sexual minorities (Gartrell et al., 1996, 1999, 2000, 2006, 2011; Goldberg, 2010). Investigators have also asked SMPs about the ways they believe their children might benefit from growing up in non-traditional families (Muzio, 1995; Lynch and Murray, 2000). However, there is a gap in the literature on the experiences of SMPs whose offspring have reached adulthood. The current study explores the most challenging and best parenting experiences of SMPs from the time that their children were conceived until they reach adulthood.

Sexual minority parenting was established as a field of study in the 1970s when lesbian mothers began to seek custody of children they had conceived in heterosexual relationships (Hunter and Polikoff, 1976; Tasker and Golombok, 1997; Tasker, 2013; Golombok, 2015). This was a time of considerable social, cultural, religious, and legal opposition to non-traditional families (Goldberg, 2010; Golombok, 2015). In the absence of empirical data on lesbian-parent families, custody was awarded to the fathers based on assumptions that children reared by lesbians would develop behavioral problems, demonstrate gender non-conformity, experience bullying, and grow up to be LGBT (Golombok and Tasker, 1996; Tasker and Golombok, 1997; Tasker and Patterson, 2008; Tasker, 2013; Golombok, 2015). Although the first research on this topic found that children raised in post-divorce lesbian-parent households were comparable in psychosocial development to those from single heterosexual-mother families (Green, 1978; Kirkpatrick et al., 1981; Golombok et al., 1983), critics pointed to the lack of prospective, longitudinal data on children, adolescents, and adults raised since birth by SMPs (Golombok, 2015). This was the climate in 1982 when The Sperm Bank of California became the first family planning clinic in the United States to offer donor insemination (DI) to all women, regardless of sexual orientation or marital status (The Sperm Bank of California, 2019).

As the news of this clinic made its way around the country, lesbian-identified prospective parents began to conceive children through DI, forming what are now known as planned (or intentional) lesbian-parent families (Gartrell et al., 1996; Goldberg, 2010; Golombok, 2015). Embarking on this path subjected this first generation of parents to criticism from their families of origin, social and religious communities, and even other lesbian women who considered the choice to parent a form of “passing” (i.e., increasing the likelihood of appearing to be heterosexual, and thus reducing one’s exposure to homophobic stigmatization), and a sell-out from the struggle for LGBTQ civil rights (Gartrell et al., 1999; Bradford et al., 2013). At the time, experts in child development, mental health, law, and public policy commented that the outcome of this new social phenomenon would only become fully apparent when the first generation of offspring conceived through DI to lesbian-identified parents reached adulthood (Kolata, 1989; Parke, 2004, 2013). These offspring have now entered adulthood in substantial numbers, providing a wealth of opportunity to study their well-being as adults, and to explore their parents’ perspectives on the bold social experiment in which they participated.

There is now an extensive body of research on the psychological well-being of children and adolescents reared in SMP families. These children and adolescents have been found to fare as well as, or sometimes better than, those raised in mother–father parent families (Gartrell and Bos, 2010). Many of these studies were grounded in family systems theory (focusing on factors that influence a child’s growth and development over time in the context of family relationships; Goldberg, 2010; Farr et al., 2017), minority stress theory (which attributes the stress of homophobic stigmatization to health disparities found among sexual minority people; Meyer, 2003), as well as social constructionism and queer theory (which emphasize that the concept of family is subject to interpretation, and view SMP families as a challenge to traditional notions of family, gender, gender relations, and sexuality; Dunne, 2000; Oswald et al., 2005; Stacey, 2006; Goldberg and Perry-Jenkins, 2007; Goldberg, 2010; Farr et al., 2017).

Few studies have examined mental health outcomes in adults who were born into SMP families (Golombok and Badger, 2010; Gartrell et al., 2018; Koh et al., 2019). The ongoing U.S. National Longitudinal Lesbian Family Study (NLLFS) began in 1986 with a goal of providing prospective data on the first generation of planned lesbian-parent families (Gartrell et al., 1996). In the sixth wave, the index children – all conceived through DI at Wave 1 by lesbian-identified prospective parents – were 25 years old. When these adult offspring were compared with same-age peers from a nationally representative sample, no differences were found in their relationships with family, friends, or spouses/partners (Gartrell et al., 2018). Also, there were no significant differences in their educational or job performance or mental health (including emotional and behavioral problems). However, NLLFS adult offspring who had been stigmatized in multiple ways because of their parents’ sexual orientation and had low scores on meaning in life were found to have higher rates of emotional or behavioral problems than the remaining NLLFS offspring (Bos et al., 2019; Koh et al., 2019).

To our knowledge, no prior investigation has explored the perspectives of SMPs on their overall parenting experiences from the time that their children were conceived until their children reached adulthood. This information is essential for future generations of SMPs as well as for professionals who may be consulted by SMP families. The current study aims to address this gap in the literature. Research suggests that first-generation DI SMPs might express concerns about the ways that homophobic stigmatization affected their children in the past or present (Gartrell et al., 1996, 1999, 2000, 2006; Goldberg, 2010). Drawing from social constructionism and queer theory (Dunne, 2000; Oswald et al., 2005; Stacey, 2006; Goldberg and Perry-Jenkins, 2007; Goldberg, 2010; Farr et al., 2017) we predict that SMPs might be proud of the ways that their sexual and gender non-conformity influenced cultural concepts of family and parenting in positive ways. The purpose of the current study was to explore the NLLFS parents’ perspectives on the entirety of their parenting, at the time when their children were 25 years old. Specifically, this investigation aimed to gain information about the parents’ most challenging and best experiences related to parenting in a non-traditional family since the time that their children were conceived.

## Materials and Methods

### Participants

One hundred and thirty one parents participated in the sixth wave of the U.S. National Longitudinal Family Study – when their offspring were 25 years old. At Wave 1 (1986–1992), lesbian-identified prospective parents who were inseminating or pregnant via donor sperm were solicited through advertisements in newspapers, flyers in women’s bookstores, and leaflets at lesbian events (Gartrell et al., 1996). Interested individuals were invited to call the principal investigator. All callers who felt that they could commit to a longitudinal study of 18+ years duration were accepted as participants. This resulted in an initial cohort of 84 families, consisting of 84 prospective birth mothers and 70 prospective co-mothers. All participants were among the first generation to conceive through DI in planned lesbian families. After Wave 1, data were collected when the index offspring were 2 years old (Wave 2), 5 years old (Wave 3), 10 years old (Wave 4), 17 years old (Wave 5), and 25 years old (Wave 6; Gartrell et al., 2018). During the sixth wave, 77 of the original families were still participating (92% retention rate). The non-participating parents included those in seven families who withdrew or were unavailable, and others who were ill or deceased (from natural causes). For the present study, two families were excluded from the analyses – one because the offspring only partially completed the survey, and another because the offspring was 26 years old at the time of survey completion. Therefore, 75 families, consisting of 69 birth parents, 55 co-parents, and 7 stepparents, were used in the current analyses.

### Procedure

After approval was obtained from Sutter Health Institutional Review Board, each NLLFS parent was contacted by email after their offspring reached the age of 25. The email explained the purpose and procedure of the study, and assured each parent that participation in the current wave, as in all prior waves, was entirely voluntary. The measures to ensure confidentiality were also explained to each potential participant (the survey would be administered through a protected online program). After informed consent was obtained and the survey was completed, each participant received a $60 gift card. Data were collected between 2012 (when the oldest offspring reached the age of 25) until 2017 (when the youngest turned 25; Gartrell et al., 2019).

### Self-Report Measures

The current study focused on two open-ended questions:

1. In the past 25 years, what was your most challenging experience related to being a parent in a non-traditional family?
2. In the past 25 years, what was your best experience related to being a parent in a non-traditional family?

### Data Analysis

With a goal of providing a descriptive account of the participants’ most salient overall experiences in non-traditional parenthood, the results were interpreted through thematic analysis (Braun and Clarke, 2006). Thematic analysis, which has been used within most theoretical frameworks, involves examining the entire data set for patterns or themes (Braun and Clarke, 2006). The research questions formed two major predetermined themes, each of which allowed for the emergence of novel categories from the participants’ responses.

#### Coding

Two members of the research team (NG and ER) read each response multiple times. These researchers then discussed the major themes that appeared in the responses to the two research questions. Subsequently, NG reread the responses eight times to create broad descriptive categories that encompassed the range of experiences reported by the participants. These categories were discussed and refined multiple times by the two researchers to create an exclusive coding scheme (one code for each phrase, sentence, or group of sentences) before conducting a practice session. NG and ER then independently coded 44 sets of responses, representing 30% of the participants. Their two independently scored sheets were submitted to the statistical analyst (a third member of the research team, HB), who calculated the Krippendorff’s alphas: 0.86 for Question 1 and 0.92 for Question 2. NG and ER discussed the responses that they had coded differently and reconciled those into a single code for each response or response segment (phrase, sentence, or group of sentences). Their high level of agreement on the Krippendorff’s alphas when coding the first 30% of participant responses made it possible for NG to code the responses of the remaining 70%.

## Results

### Descriptive Statistics

The descriptive statistics for the total analytic sample are presented in Table 1. As can be seen, 96.9% of parents identified as female (at Wave 6) and 89.3% as White (at Wave 1). Their average age at Wave 6 was 59.8 ± 4.9 years. Most parents had a college degree or higher (Wave 6; 92.4%).

**TABLE 1:** Demographic information for the sample.

	***n***	**%**
**Parent type**		
Birth parent	69	52.7
Co-parent	55	42.0
Stepparent	77	5.3
**Current gender identity**		
Female	127	96.9
Male	0	0.0
Transgender female	0	0.0
Transgender male	0	0.0
Genderqueer	3	2.3
Intersex	0	0.0
Other^1^	1	0.8
**Age (years)**		
*M* = 59.8		
SD = 4.9		
**Race/ethnicity**		
People of color^2^	7	5.3
White	117	89.3
Unknown	7	5.3
**Education**		
High school graduate or General Equivalency Diploma	2	1.5
Some college, but no college degree	8	6.1
Associate’s, bachelor’s, or registered nurse degree	23	17.6
Some graduate school, but no graduate degree	10	7.6
Masters, doctoral, or law degree	88	67.2
**Current work status**		
No work	27	20.6
Part-time	19	61.1
Full-time	80	14.5
Between part-time and full-time	5	3.8

*Birth parent and co-parent type and race/ethnicity data were collected in Wave 1, and the remaining data in Wave 6; all numbers are n (%), with exception of age. ^1^Transgender, intersex, multigender. ^2^Native American: 2, Asian American: 3, African American: 2.*

### Most Challenging Parenting Experiences

Based on the participants’ reports, 117 segments were coded. As shown in Table 2, thematic analysis revealed that the most challenging parenting experiences associated with raising their children in non-traditional families fell into five major categories: (1) distress about their children’s experiences of exclusion, heterosexism, or homophobic stigmatization (33.3%); (2) family of origin non-acceptance of their lesbian-parent family (16.2%); (3) the never-ending process of “educating the world about queer parents” (14.5%); (4) homophobia or hostility toward their non-traditional family (12.8%); and (5) lack of legal protections for SMP families (9.4%). Other challenging experiences included not having the co-mother acknowledged as a parent (6.8%), and dissatisfaction with the known donor’s role in the family (5.1%).

**TABLE 2:** Most challenging parenting experience.

	**Segment**	
	
	***n***	**%**
**Thematic coding category**		
(1) Distress about their children’s experiences of exclusion, heterosexism, or homophobic stigmatization	39	33.3
(2) Family of origin non-acceptance of their lesbian-parent family	19	16.2
(3) The never-ending process of “educating the world about queer parents”	17	14.5
(4) Homophobia or hostility toward their non-traditional family	15	12.8
(5) Lack of legal protections for SMP families	11	9.4
(6) Co-mother not acknowledged as a parent	8	6.8
(7) Dissatisfaction with the known donor’s role in the family	6	5.1
(8) Other	2	1.7
Total	117	100.0

#### Distress About Their Children’s Experiences of Exclusion, Heterosexism, or Homophobic Stigmatization

Thirty-nine parents focused on their children’s experiences of homophobic discrimination. Pat (all names are pseudonyms) described a difficult period for her son: “The most challenging experience was trying to get him through middle school years when the kids would tease him about having lesbian parents or accuse him of being gay because of it.” Samantha felt particularly bad about the ways her son was excluded: “[His] best friend had a father who disapproved of us not having a man/husband in the house. The father would not let his son stay overnight at our house and our son was never invited to his friend’s birthday parties.” Judith found it challenging “to explain the ignorance of other people to [her] kids when they were little.” Bee pointed to the difficulty of “helping <child’s name> to try to understand why a friend from a very religious environment was not allowed to come to her 12th birthday party.” Some parents felt sad or hurt when their children were ashamed about their family type as a result of having been stigmatized. Tanya wrote that it was challenging when her “son had times when he was embarrassed to have two moms or when he wouldn’t say anything when his friends made homophobic remarks in my presence.” One parent described the change in her son’s behavior after they moved from a progressive to a conservative community: “Many of the kids <child’s name> met were not exactly gay-positive, and after a few negative experiences he stopped telling his fellow students that he had two moms.” During the marriage equality debates, many parents had difficulty protecting their children from homophobic stereotypes. Amelia said that “the hardest part was that our daughter was subjected to hearing mean-spirited things about her own family, also at school, in our community, and in the media.” Nicola and Jane “were concerned with [their] daughter’s well-being and safety as [they] began to speak out publicly about [their] family and [their] desire to marry.”

#### Family of Origin Non-acceptance of Their Lesbian-Parent Family

Nineteen participants reported that they had been rejected by their family of origin for choosing to raise a child in a lesbian-parent household. Penny wrote, “Just about the time I became pregnant, my…brother and his wife became born again Christians. My brother said some very hurtful things to us. For a couple of years after my son’s birth, we would never allow my brother and sister-in-law to be alone with him. We didn’t know if they would kidnap him or make derogatory comments about us to him.” Jessica stated, “My greatest difficulty has been with my own original family, some of whom have found it nearly impossible to accept me as a lesbian, and as a result of that, accept my partner and my children as normal. I have tried for years to show up with my family and participate in the larger circle. But some, I guess because of deep religious conviction, cannot let us be normal and part of the whole. It’s so disappointing.” Alexandria, a birth mother, stated that her co-parent Rachel had lost custody of a child due to homophobia. Rachel’s family of origin had used the legal system to claim that Rachel was unfit to parent because she was a lesbian: “They were instrumental in her losing custody of her then 4-year-old daughter because we were a lesbian couple.” Some parents indicated that their families of origin had been hostile when the pregnancy was announced, but eventually came around. Bev described the painful experience of her “parents telling [her] that <child’s name> was not their grandchild. They changed within a couple of years and were very loving and proud, but it took some work.” For others, the rejection by the family of origin never ceased. Kim wrote, “My biological family was/is a great challenge. They rarely recognize my children for holidays, etc.” Alice stated, “My parents never accepted our kids as their grandchildren because they were children of lesbians.” Celia’s mother took her homophobia to the grave: “At my mother’s shiva 6 years ago, a woman came up to our daughter and said, ‘I am so glad your mother decided to have you. I remember your grandmother and mother had talked about whether she would go through with the pregnancy – I’m so glad she decided to do so.’ My daughter came to me and literally said ‘WTF!’ I explained that my mother had told people I was pregnant by accident but had then decided to have the baby. She told people I was a single parent when in reality I had been in a lesbian relationship (family) for 25 years.”

#### The Never-Ending Process of “Educating the World About Queer Parents”

Seventeen parents found the necessity of repeatedly having to explain their family configuration annoying. As Cecilia put it, “We were among the first non-traditional families, so other parents were not sure what to make of us.” Debbie tired of “the relentlessness of having to explain we are both parents…[and] having people ask, ‘What do you know about the donor?’ UGH.” One participant mentioned that the conversation became even more complex when she and her co-parent split, and her daughter then had two moms and a stepmom: “I did have to talk to every single teacher for both girls every year to let them know that they had two moms and a stepmom, and that <child’s name> lived in two homes.” Another parent referred to it as the “many moms” conversation.

#### Homophobia or Hostility Toward Their Non-traditional Family

Dealing with homophobia or hostility that was directed at their families was particularly challenging for 15 parents. Some cited early incidents that were traumatic. Barb stated, “When my son was 2 years old, someone I supervised at work organized an attack on me and told everyone I was a lesbian and tried to get me kicked out of my job. Although I didn’t lose my job, this was really scary. I felt a strong sense of being vulnerable as a lesbian mom and remember feeling scared that my son could be taken away from me. The trauma of that experience, although greatly healed, is still with me.” Cassie wrote that she felt that “it was important to be ‘out’ for [her] child, but [she] often felt some fear while doing so.” Several mentioned homophobic experiences in the medical setting. Valerie hated “having to deal with doctors and hospitals that did not recognize [her] right to parent [her] kids.” Kaye Jean elaborated on one such experience: “At <name of hospital>, we walked in the room to meet the head doctor for the first time. She did not introduce herself. She immediately yelled, ‘We have to know about the father.’ We were shocked by this unprofessional approach. I would have expected her to start by asking us for a family medical history.” When her child was very young, Jasmine had to contend with a complaint filed with social services by a neighbor: “It was very challenging, but the support we got from the pre-school, the pediatrician, other neighbors and friends was incredible.” Several participants described their efforts to destigmatize SMP families by befriending opponents. Danielle wrote, “There was one mother who was very rude, very uncomfortable around my wife and I. I chose to volunteer in the library every week, the same day as this mother. Within 6 months…she started to talk about being more accepting of a non-traditional family. She did not know any other lesbian or gay people. I think she just had a preconceived idea (with a little influence from her Catholic upbringing) that we were bad people.” Wendy felt that cultural homophobia contributed to the dissolution of the relationship with her co-parent: “I sometimes wonder if we could have kept our family intact if we had been a traditional couple. I do see straight couples under stress and imagine more social/societal support to stay together than we had.”

#### Lack of Legal Protections for SMP Families

Eleven participants pointed to the lack of legal protections for their families, especially before marriage equality was granted by the United States Supreme Court. Some of these participants were litigants in the struggle for co-parent adoption. Marty stated, “<Co-mother’s name> and I were the first <county name> citizens to legally adopt our non-biological children and have both our names listed on their birth certificates. At the time, the <state name> only allowed adoptions by a man and a woman. The process was expensive, time-consuming, and sometimes frustrating.” Silvana described her family’s struggle to obtain medical insurance for their child before co-parent adoption: “Before we were able to cross-adopt, when <child’s name> was born, I argued she should go on my insurance, because she was my dependent. After agreeing, this option was withdrawn. It made me angry that my parenthood was being written out of existence, and anxious because <child’s name> had some serious medical problems.” Several mentioned the stress of traveling out of state and the prospect of officials denying the legitimacy of their family ties. Roberta wrote about “not being recognized as a family by the government and always having to be sure you had all the legal paperwork complete and up-to-date at all times.” Sallyanne stated, “We were nervous traveling to Texas, that if something happened, they would take <daughter’s name> since her family was not recognized. It was insulting for my partner to need to adopt her own child.” Some co-parents, like Pauletta, described being fearful when she broke up with her children’s birth mother “that the courts would [not] honor my role in my children’s lives when we split as I was not the biological mother.”

#### Dissatisfaction With the Known Donor’s Role in the Family

Dissatisfaction with the role of the known donor was mentioned by six participants. A few wished that the donor had been more involved in their family life, and others wanted the opposite. Andrea wrote: “Although we have a known donor, I feel very sad that we rarely see him (every few years at this point), and almost never unless I initiate.” In contrast, Karen regretted “having to send [her] child (beginning at 6 months of age) to her father’s home on alternate weekends during the week, and for extended periods in the summer.”

### Best Parenting Experiences

Based on thematic analysis, 107 segments were coded from the participants’ recollections of their best experiences as parents in non-traditional families over the prior 25 years (see Table 3). Four primary categories emerged from these codes: (1) being role models, leading to a greater acceptance of LGBTQ people (23.4%); (2) treasuring the LGBTQ parent and family community (21.5%); (3) teaching their children to appreciate diversity of all types (19.6%); and (4) witnessing their child’s pride in their non-traditional family (18.7%). Other themes that were associated with best experiences included gaining legal recognition (9.3%), and having the freedom to parent across gender expectations (3.7%).

**TABLE 3:** Best parenting experience.

	**Segment**	
	
	***n***	**%**
**Thematic coding category**		
(1) Being role models, leading to greater acceptance of LGBTQ people	25	23.4
(2) Treasuring the LGBTQ parent and family community	23	21.5
(3) Teaching their children to appreciate diversity of all types	21	19.6
(4) Witnessing their child’s pride in their non-traditional family	20	18.7
(5) Gaining legal recognition	10	9.3
(6) Having the freedom to parent across gender expectations	4	3.7
(7) Other	4	3.7
Total	107	100.0^1^

*^1^Due to rounding, the total is 99.9%.*

#### Being Role Models, Leading to Greater Acceptance of LGBTQ People

Twenty-five parents were proud to have shown people in all walks of life how healthy, loving, and supportive SMP families could be. They described their delight in witnessing greater acceptance and recognition of LGBTQ-parent families. Many commented on their efforts to educate people about their family type. Deni stated, “I enjoyed…educating other lesbian women who wanted to parent. I liked challenging people’s assumptions about who makes a good parent and why.” Sondra wrote, “In the beginning it was extremely hard, but as we moved forward, you could see a shift in how people viewed our family. It really was something wonderful to see.” Kim reported on a school experience: “I loved it when our son’s third grade teacher told us how worried she had been to have lesbians be part of the parents she had to deal with at the beginning of the school year, and how unnecessary that worry had been – that we were the best parents in the room.” Some, like Dale, wrote about “coming out to people in my work or in my social circles over and over and seeing their views change over time, going from either neutral or ignorant to educated and supportive of gay and lesbian parenting.” Some parents mentioned past and present opportunities to mentor youth. Ann stated, “For straight friends, I have been someone their kids could come talk to.” Others were still receiving feedback about how helpful they had been. Cassie wrote, “In recent years we’ve heard from some of our daughter’s friends – those who are grown up now and out as gay, lesbian, and trans – that growing up they looked up to us as role models and that we gave them some hope that everything could work out.” Libby added, “We have been able to mentor several lesbians, gays, and transgender youth in our community – those who now know that non-traditional families can live happy, productive lives, and have children.”

#### Treasuring the LGBTQ Parent and Family Community

For 23 parents, the LGBTQ community was a highlight of their parenting years. Many commented on the important connections they had made during LGBTQ Pride celebrations, family camps, and community events. Francis mentioned that she “feel[s] a deep connection to the LGBTQ parenting world.” Erin described her favorite memory: “Blueberry Cove Family Camp, with all kinds of families, and having a week of bliss each summer for many years, during which we were just another family that had two wonderful moms and two wonderful sons and many, many good, loving friends to eat with, swim with, write with, play with, and the kids could be freeeeeee.” Joan’s family still goes to queer family camp every year: “Camp it Up, a family camp for queer families, we go every summer. It’s an essential recharge each year, to have our family affirmed and reflected in a safe environment.” Several parents mentioned Family Week in Provincetown as a special annual event. Some also included their religious communities as sources of support. Marie stated, “We have had a great deal of support and affirmation as a lesbian family in our community – synagogue, family camp, community of friends, and family.”

#### Teaching Their Children to Appreciate Diversity of All Types

The best parenting experience for 21 participants involved teaching their children to appreciate all forms of diversity and understand all forms of oppression. Margarita captured this concept in her response: “The best experience was helping to raise an incredible son. He is a sensitive, caring, loving, compassionate person because of the family he was born into, with two great mothers and two great fathers. All of his parents have done a good job of parenting. Being from a gay family has contributed to his understanding of all oppressions – racism, sexism, classism, and gay oppression. He has been a great ally to me, particularly as a woman of color who was raised poor from an immigrant family.” Shay wrote of her pride in “watching the ways that both my children actively care for the disadvantaged and stand up for the rights of everyone to equal treatment and opportunity.” Ansley stated, “I always knew from a very young age that any children I brought into this world would be loved, healthy, happy, productive individuals who give back to the world. This has proved to be 100% true.” Several parents, like Clare, mentioned their joy in raising empathic sons: “Being able to raise <child’s name> with an open mind to different ways of living, nurturing [his] ability to be empathic and sensitive to others.” Others commented that helping their children appreciate diversity had the additional benefit of giving them more confidence in themselves, as Maddy’s response illustrates: “I believe that our children have learned to be accepting of all types of families and people in general. From a very young age, they learned to stand up for themselves and who they are.” Danny mentioned that she was very happy in “seeing that our role modeling and community helped our kids understand that they could be truly themselves, in whatever way they turned out to be.” She was also grateful for “their having an appreciation of otherness and difference that helped them be more accepting of diversity in others.”

#### Witnessing Their Child’s Pride in Their Non-traditional Family

Twenty parents wrote about witnessing their child’s pride in their non-traditional family. Many described important events at which their children spoke or taught others about their family type. Sal commented on an occasion when her young son educated prospective SMPs: “He and some of his friends spoke on a panel at an event for lesbians considering parenthood. [It] was pretty cool too, how positively he expressed he felt about growing up in a lesbian family.” Jovi described a proud moment when her son’s essay educated his entire school: “When my son was in elementary school (5th grade), he wrote a paper about why he was disappointed in the State of California for trying to pass a law about not allowing same-sex couples to marry (Prop 22). His teacher thought it was a wonderful paper and asked the principal if he could read it to the entire school during the assembly that day. So many of the parents came up to us to let us know that hearing his paper had changed their view on their vote. They had not thought about how unfair it was to not have the same rights that [were] allowed to others.” Francis wrote that her children were invited to be panel participants at an annual event for social workers: “They were asked to speak about what it had been like being raised by same-gender parents. It felt like an honor.” Others had children who testified for LGBTQ civil rights. In doing so, Diane stated that her daughter was “very mature and comfortable with who she was, even at the tender age of 13!” Sue wrote that her son has made it his life’s work to educate the world about his non-traditional family: “He is a writer who has taken many of his childhood experiences and turned them into entertaining and enlightening stories, some of which he has told on radio programs and in community story-telling programs. I am proud of how he has learned to reframe his experiences, to take ownership of the out-of-the-mainstream experiences he has had, and to share his perspective with others through his creativity.”

#### Legal Recognition

For 10 parents, the highlight of their parenting experiences was gaining legal recognition through the United States Supreme Court decisions. Cece was proud of “being involved in the equal marriage litigation as a family.” Sheila wrote that her best experience was “getting married because of the legal security for us, but [also] getting married because the children really wanted us to.”

#### Freedom to Parent Across Gender Expectations

Four parents felt that it was particularly important that they were able to parent across gender expectations. As Jaime put it, “At our house, there were no real traditional gender roles, so anything was seen as possible to do. As a result, my daughter is very sensitive to people saying something like, ‘You can’t do it because you’re _______.”’

## Discussion

This study is unique in examining the perspectives of SMPs whose children are now emerging adults. These parents were among the first generation to conceive children through DI and rear them in planned lesbian families. The parents were participants in the sixth wave of the ongoing NLLFS. They were asked to describe their most challenging and best experiences as parents in non-traditional families from the time that their children were born until they reached the age of 25 years. Thematic analyses revealed that the most challenging experiences faced by the NLLFS parents included distress about their children’s experiences of exclusion, heterosexism, or homophobic stigmatization; family of origin non-acceptance of their lesbian-parent family; the never-ending process of educating the world about SMPs; homophobia or hostility toward their non-traditional family; and lack of legal protection for SMPs. The best experiences fell into four main categories, namely, serving as role models who contributed to a greater acceptance of LGBTQ people, treasuring the LGBTQ parent and family community, teaching their children to appreciate diversity of all types, and witnessing their child’s pride in their non-traditional family.

Studies on SMP families in the transition to parenthood or the early years of childhood (Gartrell et al., 1996, 1999, 2000, 2006; Goldberg and Sayer, 2006; Goldberg, 2010) might have predicted that even as parents of adult children, the NLLFS participants would comment on their children’s experiences of homophobic stigmatization. As it turned out, this was the most frequently mentioned parenting challenge by participants in the current study, and it was anticipated by the NLLFS parents at Wave 1 (Gartrell et al., 1996). At that time, the prospective parents worried that their children could be stigmatized because they had been conceived through DI by lesbian-identified women. In preparation for helping their children cope with this challenge, 61% of prospective parents had formed or joined lesbian parenting groups. After their children were born, the parents were actively involved in promoting LGBTQ-awareness in their pre-schools, elementary schools, social groups, and community activities (Gartrell et al., 1999, 2000). The NLLFS parents felt that exposure to all types of diversity was essential to protecting their children from homophobia (Gartrell et al., 1999), and many parents taught their children healthy responses to harassment (Gartrell et al., 2005). Since that time, studies have found that promoting awareness of diversity and preparing for the prospect of discrimination are important aspects of cultural socialization for non-majority children (Hughes et al., 2006; Oakley et al., 2017). When the NLLFS offspring were 10 years old, 43% reported that they had experienced discrimination based on their family type, and nearly 40% spoke out about it, telling peers that they were “wrong” or “not nice” for making hostile, homophobic comments (Gartrell et al., 2005). The negative impact of homophobia on the psychological well-being of the NLLFS offspring who experienced it was moderated (or lessened) for 10-year-olds who attended schools with LGBTQ curricula and had parents who participated in the lesbian community (Bos et al., 2008); for 17-year-olds, by having close, positive relationships with their parents (Bos and Gartrell, 2010); and for 25-year-olds who had found meaning in life (Bos et al., 2019).

In the current study, for many NLLFS parents, rejection by their family of origin was still a salient memory. At Wave 1, 15% of participants expected that no family member would acknowledge their child because of their own lesbian identity (Gartrell et al., 1996). However, by the time the index offspring were 10 years old, most families of origin had embraced these children and treated them no differently than any other family members (Gartrell et al., 2006). Also, nearly three-quarters of grandparents were “out” about their grandchild’s SMPs. Nevertheless, some parents in the current study had family members who never accepted the index offspring or the SMP family in which they were raised.

Educating others about non-traditional families was a never-ending process for many parents in the first generation of lesbian-identified women to conceive children through DI. When their children were young, these parents had to contend with considerable cultural and institutional homophobia (Gartrell et al., 1996; Tasker and Patterson, 2008; Goldberg, 2010; Bradford et al., 2013; Golombok, 2015). The struggle to create a safe path for their children was at times arduous, according to parent reports across the six waves of the NLLFS (Gartrell et al., 1996, 1999, 2000, 2005). For example, whenever their children changed schools, joined an athletic team, or enrolled in community theater, the parents had to come out to a new group of families, teachers, coaches, or instructors. This meant monitoring to ensure that their children were not stigmatized, and if they were, working to promote acceptance of non-traditional families. In two-mother families, there was an ongoing effort to ensure that both parents were acknowledged as legitimate. In order to do so, many parents in this first generation helped forge the legal protections that SMPs now enjoy. All in all, first-generation SMPs contributed to cultural and institutional changes in the acceptance of non-traditional families in ways that could not have been anticipated when they first embarked on the path to parenthood.

A quarter century after this bold experiment began (Parke, 2004, 2013), the NLLFS parents were proud of their role in promoting greater acceptance of LGBTQ people. In line with social constructionism and queer theory, their best parenting experiences included challenging heterosexual norms about sexuality and gender (Oswald et al., 2005) and what it means to be a family (Dunne, 2000; Oswald et al., 2005; Stacey, 2006). They described special memories of joyful celebrations and connections with the LGBTQ parent and family community. They appreciated that their adult children welcomed diversity, were unrestricted by gender stereotypes, and continued to educate their own peers about non-traditional families.

### Strengths, Limitations, and Future Directions

A strength of the current study is that the data were drawn from the largest and longest-running, ongoing, longitudinal investigation of SMPs. Because the NLLFS is a prospective investigation, the findings from the current study are not biased by overrepresentation of parents who volunteered to participate when they already knew that their families and children were doing well.

There are also limitations that must be noted. The NLLFS is a non-representative sample. The parents enrolled at a time in history when most LGBTQ people were closeted, and recruiting a population-based sample was unrealistic. In addition, the participants were mostly White and highly educated. For these reasons, the results may not be generalizable to the population of SMPs as a whole.

Future prospective, longitudinal studies would benefit from larger, more diverse, and representative samples of parents who identify as LGBTQ, for whom children entered the family unit through DI, fostering, adoption, stepparenting, and surrogacy. These samples would allow for an intersectional approach to explore the parenting experiences of a wide array of sexual and gender minority people, including those who have multiple minority status, in the post-marriage equality era. Because the current study asked parents to reflect on their experiences over a 25-year period, some reported on past and some on recent events. Research is needed to assess whether societal and cultural changes during this period of time have created more of a welcoming environment for newly forming SMP families, or whether these changes are only affecting those who reside in more progressive communities or countries.

Our findings provide implications for practice. Health professionals, educators, and social service agents should be attentive to the prospect that SMPs and their children may be subjected to homophobic discrimination from many sources – families of origin, medical and mental health professionals, teachers, peers, and colleagues. Clinicians should be prepared to help SMPs and their families manage the stress of coming out over and over, sometimes to individuals who may be hostile. Clinicians and educators should also understand the importance of preparing the children of SMPs for the prospect of stigmatization. SMPs should work with the school systems to design or improve anti-bullying programs, and to ensure that the educational curricula provide information on all types of families (Gartrell et al., 1999, 2000, 2005; Hughes et al., 2006; Oakley et al., 2017). In addition, professionals should be fully informed about the favorable outcomes and protective factors for children reared in SMP families (Bos et al., 2008, 2019; Bos and Gartrell, 2010) so that they can share this information with prospective or struggling parents. Despite the many challenges faced by the first generation of SMPs who conceived through DI, their adult children are faring very well (Golombok and Badger, 2010; Gartrell et al., 2018).

## Conclusion

To our knowledge, this is the only study to have surveyed SMPs about their most challenging and best parenting experiences from the time that their children were conceived until they became 25-year-old adults. The results revealed that distress over their children’s and family’s experiences of homophobic stigmatization, family of origin non-acceptance of their lesbian-parent family, the never-ending process of educating people about non-traditional families, and lack of legal protections for SMPs families were the most challenging experiences recalled by the participating parents. On the positive side, the parents were proud that they had contributed to the greater acceptance of SMP families, and that they had taught their children to welcome diversity. They treasured their memories of connecting with the LGBTQ parent and family community, and witnessing their children speak publicly and favorably about their non-traditional family.

## Data Availability Statement

The raw data supporting the conclusions of this manuscript will be made available by the authors, without undue reservation, to any qualified researcher.

## Ethics Statement

The studies involving human participants were reviewed and approved by Sutter Health Institutional Review Board. The patients/participants provided their written informed consent to participate in this study.

## Author Contributions

All authors designed the study and made substantial intellectual contributions to the work. HB managed the data file and conducted the statistical analyses. NG and ER developed the thematic coding scheme and coded the responses. NG took the lead in writing the manuscript. All authors revised the manuscript and approved it for publication.

## Conflict of Interest

The authors declare that the research was conducted in the absence of any commercial or financial relationships that could be construed as a potential conflict of interest.
